# Classism, Perceived Stress, and Mental Health Symptoms: Cross-Sectional Evidence from a Census-Matched U.S. Sample

**DOI:** 10.3390/healthcare14091205

**Published:** 2026-04-30

**Authors:** David G. Figueroa, Monica Chen, Matthew Phillipi, Jordan E. Parker, Jeffrey M. Hunger, A. Janet Tomiyama

**Affiliations:** Department of Psychology, University of California, Los Angeles, Los Angeles, CA 90095, USA; dfigueroa@ucla.edu (D.G.F.); matthewphillipi@gmail.com (M.P.);

**Keywords:** classism, perceived stress, depression symptoms, anxiety symptoms, social class

## Abstract

**Background/Objectives:** Classism, or discrimination based on social class, is associated with higher levels of depression and anxiety symptoms. However, limited research has examined the psychological processes that may help explain these associations. The present study tested whether perceived stress statistically mediated the associations between experienced and anticipated classism and mental health symptoms. **Methods:** A U.S. census-matched sample on age, gender, race/ethnicity, income, and census region (*n* = 1993) was analyzed. Missing data were addressed using Bayesian multiple imputation, and mediation models estimated total, direct, and indirect effects. **Results:** Results indicated that perceived stress statistically accounted for the associations between both experienced and anticipated classism and higher depression and anxiety symptoms, even after adjusting for income and education. In exploratory analyses, individuals living at or below the federal poverty line reported a higher likelihood of experiencing classism, and perceived stress significantly mediated the association between experienced classism and mental health symptoms within this population. **Conclusions:** These findings provide preliminary evidence that perceived stress is a statistical mediator of the association between classism and mental health symptoms. Future prospective and experimental work is required to establish potential causal relationships between the constructs.

## 1. Introduction

Social class is a powerful social determinant of health that shapes both risk of poor health and exposure to stigma and discrimination. Individuals may be treated poorly or devalued based on how others perceive their social class, a form of discrimination referred to as classism [1,2]. Similarly to other forms of discrimination, classism can be both directly experienced in interpersonal interactions and anticipated in the form of concern or fear about future class-based mistreatment [3]. Classism has been linked to poorer psychological well-being, including higher levels of depression and anxiety [4,5]. Despite growing evidence linking classism to mental health outcomes, less is known about the psychological mechanisms through which class-based discrimination may contribute to mental health symptoms.

One possibility is that experiences of classism are appraised as psychologically stressful. Perceived stress reflects the extent to which individuals evaluate their lives as unpredictable, uncontrollable, and overwhelming [6]. The Stigma-Induced Identity Threat Model [7] provides a useful framework for understanding why stigmatizing and discriminatory experiences may have negative psychological consequences. According to this model, stigma-related stressors can threaten an individual’s social identity by signaling social devaluation or marginalization. Such identity threats may prompt individuals to appraise situations as threatening or overwhelming, thereby triggering cognitive, emotional, and physiological stress responses. Over time, repeated exposure to identity-threatening experiences may accumulate and contribute to adverse mental health outcomes [8]. Social class can be a central aspect of social identity [9] and experiences of classism may thus represent identity threats that can contribute to psychological stress.

Although literature within classism is limited, evidence from the broader discrimination literature supports the proposed study’s stress mediation hypothesis. Seminal work examining racism and health among African Americans conceptualizes perceived stress as a key psychological process through which discriminatory stressors may influence health and well-being [10]. Consistent with this perspective, meta-analytic evidence indicates that perceived discrimination is associated with both greater psychological stress and poorer mental health outcomes, even after accounting for demographic factors [11,12,13]. Although emerging evidence suggests that classism is associated with higher levels of perceived stress [14], limited research has directly examined whether psychological stress processes help explain the relationship between classism and mental health symptoms. The present study addresses this gap in the literature by testing whether perceived stress mediates the association between classism and mental health symptoms in a census-matched sample of adults in the United States. We hypothesized that higher levels of experienced and anticipated classism would be associated with higher perceived stress, which in turn would be associated with higher depression and anxiety symptoms.

Because economic disadvantage is associated with both greater exposure to discrimination and increased vulnerability to poor health outcomes [15], it is also important to further examine these pathways within the context of poverty. Poverty involves severe economic and resource deprivation and is associated with disproportionately high rates of poor mental health [16,17]. Individuals living in poverty may also encounter more frequent class-based discrimination. Longitudinal evidence from the Midlife in the United States study indicates that class-based discrimination has increased over time, with individuals at the lowest socioeconomic levels reporting substantially greater discrimination in the 2010s compared with the 1990s [2]. This evidence suggests that social class may function as an increasingly salient stigmatized identity with important implications for mental health among economically disadvantaged individuals. Examining these associations within a subsample of individuals living at or below the federal poverty line may therefore provide insight into the stress-related processes through which classism contributes to mental health disparities among this marginalized group. Thus, we additionally tested the hypothesis that higher perceived stress would mediate the relationship between classism and mental health symptoms among individuals living at or below the federal poverty line.

## 2. Methods

### 2.1. Sample

Participants (*n =* 2022) were recruited for a *Qualtrics* (Provo, UT; version accessed 1/12/2019) panel administered between December 2019 and January 2020. The sample was census-matched on age, gender, race/ethnicity, income, and census region. The census-matched sample was achieved using quotas. Participants were considered eligible if they were over 18 years old and were English-speaking. Responses were excluded if one or more of the following conditions were met: failed the attention check; reported implausible height (<44 inches or >90 inches) or weight (<55 pounds or >1000 pounds); and implausible BMIs less than 12 or greater than 70. These exclusion criteria were applied as part of the broader parent study’s data quality procedures and were not specific to the aims of the current analyses. The final analytic sample consisted of 1993 respondents. Table 1 displays sample characteristics. The sociodemographic composition of the sample was closely aligned with available U.S. census benchmarks; accordingly, survey weights were not applied in the analyses. All procedures were approved by the university’s institutional review board.

### 2.2. Measures

#### 2.2.1. Experienced Classism

Experienced classism was assessed using an item adapted from Williams and colleagues [18]. Participants were asked how often they were treated with less respect, harassed, or discriminated against because of their social class on a 4-point scale, with a higher score reflecting greater experienced classism. This item was selected to capture direct interpersonal experiences of classism. The measure was included as part of a larger survey and a single-item format was used to reduce participant burden.

#### 2.2.2. Anticipated Classism

Anticipated classism was assessed using an item modeled from Hunger and colleagues [19]. Participants were asked how often they were concerned about or worried that they would be negatively stereotyped or mistreated because of their social class on a 4-point scale, with a higher score reflecting greater anticipated classism. This item was intended to capture the fear of future classism, even in the absence of direct experiences. Similarly to experienced classism, a single-item format was used to minimize participant burden.

#### 2.2.3. Depression Symptoms

Depression symptoms were assessed using the 4-item Patient Reported-Outcomes Measurement Information System (PROMIS) depressive symptoms short form (e.g., “Little interest or pleasure in doing things” [20]. Participants reported depression symptoms over the last 7 days on a 4-point scale (Not at all–Nearly Every Day). Higher scores reflected greater depression symptoms (*α* = 0.94).

#### 2.2.4. Anxiety Symptoms

Anxiety symptoms were assessed using the 4-item PROMIS anxiety short form (e.g., “I found it hard to focus on anything other than my anxiety” [21]. Participants reported anxiety symptoms in the last 7 days (Not at all–Nearly every day). Higher scores reflected greater levels of anxiety (*α* = 0.93).

#### 2.2.5. Perceived Stress

Perceived stress was assessed using a modified 4-item Perceived Stress Scale (e.g., “How often have you felt nervous and stressed?” [6]. Participants reported perceived stress over the past month on a 5-point scale (Never–Very Often). Higher scores reflected greater levels of perceived stress (*α* = 0.87).

#### 2.2.6. Sociodemographics

Demographic variables (i.e., age, race/ethnicity, gender, census region) were included as covariates. Age was reported in years. Race/ethnicity, gender, and census region were assessed using multicategorical items. Annual household income and the highest level of education were also included as covariates to test classism independently of objective socioeconomic status (SES). Annual household income was assessed using a 7-point ordinal item ranging from less than $25,000 to more than $200,000. Level of education was assessed using a multicategorical item and was collapsed into three categories for analyses. These covariates have been used in prior research analyzing the same sample [3].

A binary poverty status variable was created for analyses among a subsample of individuals living at or below the federal poverty line. Income categories were converted to approximate midpoint income values and compared with the 2019 U.S. Department of Health and Human Services federal poverty thresholds corresponding to self-reported household size. Participants whose estimated income fell at or below the poverty threshold were coded as 1 (at or below the federal poverty line), and those above the threshold were coded as 0 (above the federal poverty line).

### 2.3. Statistical Analyses

#### 2.3.1. Confirmatory Analyses

Zero-order correlations were first computed among predictor, mediator, and outcome variables using the complete dataset in RStudio (version 2025.05.1+513). Mediation models were also estimated using the rBlimp package 0.2.25 [22]. A single model simultaneously included experienced classism and anticipated classism as predictors, perceived stress as the mediator, and depression and anxiety symptoms as outcomes. Given the high correlation between experienced and anticipated classism in this sample (r = .73), including both predictors in the same model allowed for estimation of their unique associations with perceived stress and mental health outcomes, independent of their shared variance. Residuals for depression and anxiety symptoms were allowed to correlate to account for their shared variance. Models adjusted for demographic and socioeconomic covariates, including age, household income, race/ethnicity, gender, census region, and educational attainment. Race/ethnicity, gender, census region, and education were automatically dummy coded in rBlimp, with Asian race, male gender, Northeast region, and high school or less/some college as the reference groups, respectively. Inspection of cross-tabulations for race/ethnicity and education indicated low-frequency category combinations. Accordingly, these variables were specified as “sparse” predictors to reduce the number of estimated parameters and improve model convergence. Any missing data were addressed using Bayesian multiple imputation implemented in rBlimp, which allowed for the incorporation of uncertainty due to missingness into parameter estimates and reduced bias introduced by listwise deletion. Imputation and model estimation were conducted using two Markov Chain Monte Carlo (MCMC) chains with 10,000 burn-in iterations followed by 10,000 posterior iterations. Convergence was assessed using potential scale reduction diagnostics, which compared parameter distributions across the two MCMC chains [23]. Potential scale reduction values below 1.05 indicated adequate convergence. The indirect effect was calculated as the product of the regression coefficient for the association between classism and perceived stress and the coefficient for the association between perceived stress and mental health symptoms. The indirect effect quantified how much of the association between classism and mental health symptoms is statistically explained by perceived stress. Total effects were calculated as the sum of the direct and indirect effects. An effect was considered significant if the 95% credible interval did not include zero. The proportion mediated was calculated by dividing the indirect effect by the total effect. The effect size was estimated using the squared standardized indirect effect [24]. The study measures, hypotheses, and analytic plan for confirmatory analyses were pre-registered (https://osf.io/65qk9/overview?view_only=03fd2eb730734f3887b76a6b6c9926dc; accessed 10 July 2023).

#### 2.3.2. Exploratory Analyses

A subsample was created from the full dataset including individuals meeting the federal poverty threshold (*n* = 301) for exploratory analyses. Post hoc descriptive analyses were conducted to examine whether exposure to classism differed by poverty status. Logistic regression models were estimated to test whether living at or below the federal poverty line predicted the likelihood of reporting experienced classism and anticipated classism. For these analyses, classism variables were dichotomized such that individuals who reported never experiencing classism were coded as 0 and those reporting any level of classism were coded as 1. Poverty status was entered as the primary predictor. Models adjusted for the same demographic and socioeconomic covariates included in the primary analyses, including age, race/ethnicity, gender, census region, and educational attainment. Income was not included as a covariate as it was used to calculate the poverty variable. Odds ratios and predicted probabilities of reporting experienced and anticipated classism by poverty status were estimated. Furthermore, the focal hypothesis that perceived stress would statistically mediate the association between classism and mental health outcomes was examined among this subgroup. The same modeling approach described for the focal model was used. Experienced and anticipated classism were included simultaneously as predictors, perceived stress as the mediator, and depression and anxiety symptoms as outcomes with correlated residuals. Missing data were handled using the same Bayesian multiple imputation procedure with 10,000 burn-in iterations and 10,000 posterior iterations across two Markov Chain Monte Carlo chains. Effects were considered statistically significant if the 95% credible interval did not include zero. Exploratory analyses were not pre-registered.

## 3. Results

### 3.1. Confirmatory Results

Zero-order correlations and descriptive statistics for all variables of interest are presented in Table 2. Both experienced and anticipated classism were positively correlated with depression symptoms, anxiety symptoms, and perceived stress. Perceived stress was positively correlated with depression and anxiety symptoms. Living at or below the federal poverty line (vs. above) was positively correlated with all focal variables. Table 3 presents the results from the mediation model with the complete sample. Potential scale reduction diagnostics indicated the two MCMC chains successfully converged at the end of the burn-in period, indicating that parameter estimates were stable across chains. Confirmatory analyses indicated that both experienced and anticipated classism were positively associated with higher perceived stress, and higher perceived stress was associated with higher depression and anxiety symptoms. The indirect pathway through perceived stress statistically explained between 25% and 54% of the association between classism and mental health symptoms. The proportion of variance in mental health symptoms explained by perceived stress was small (υ = 0.01 to 0.03) per Cohen’s benchmarks [25].

### 3.2. Exploratory Results

**Figure 1:**
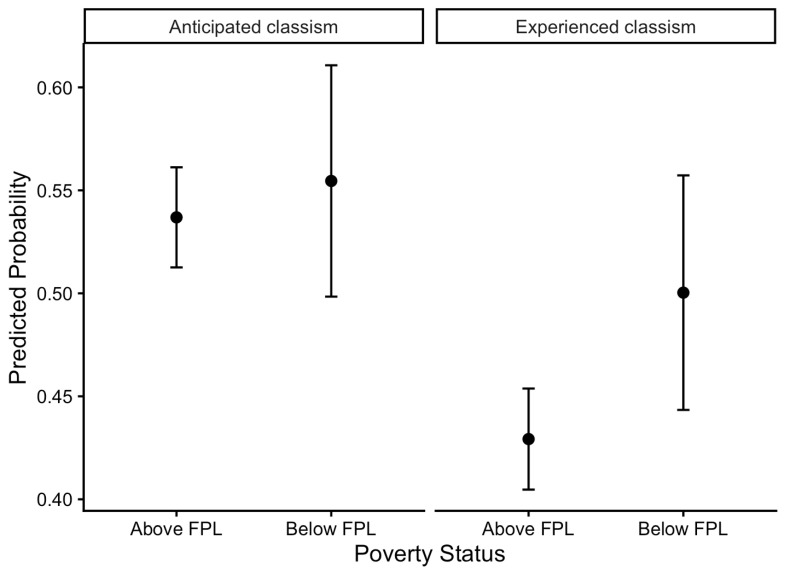
Predicted probability of anticipated and experienced classism by poverty status. *Note.* Predicted probabilities were estimated from logistic regression models examining whether poverty status (living at or below the federal poverty line vs. above the poverty line) predicted reporting any experienced or anticipated classism. Models adjusted for age, race/ethnicity, gender, census region, and educational attainment. These analyses were conducted *post hoc* and should be interpreted as exploratory.

Exploratory analyses examined whether the likelihood of reporting classism differed by poverty status. Given the smaller subsample size (*n* = 301) and the inclusion of multiple covariates and indirect effects, these analyses may be underpowered and should be interpreted as preliminary. Logistic regression models estimated predicted probabilities of reporting experienced and anticipated classism among individuals living above versus at or below the federal poverty line (Figure 1). Individuals living at or below the federal poverty line had significantly higher odds of reporting experienced classism compared with individuals living above the poverty line, *OR* = 1.37, 95% CI [1.04, 1.73], *p* = 0.026. In contrast, poverty status was not significantly associated with anticipated classism, *OR* = 1.08, 95% CI [0.82, 1.44], *p* = 0.577. Additional exploratory analyses tested the focal mediation hypothesis among a subsample of individuals living at or below the federal poverty line. Table 4 displays the results from the mediation model with the poverty subsample. Potential scale reduction diagnostics indicated that the MCMC chains successfully converged by the end of the burn-in period. Among individuals living at or below the federal poverty line, perceived stress significantly mediated the association between experienced classism and higher depression and anxiety symptoms. Specifically, higher levels of experienced classism were associated with higher levels of perceived stress, which in turn was associated with higher depression and anxiety symptoms. Perceived stress accounted for 38% to 54% of the relationship between experienced classism and mental health symptoms. The proportion of variance in mental health symptoms explained by perceived stress among individuals living at or below the federal poverty line was small (υ = 0.01). In contrast, the indirect effects linking anticipated classism to depression and anxiety symptoms through perceived stress were not significant.

## 4. Discussion

This study tested the hypothesis that the association between classism and depression and anxiety symptoms would be statistically mediated by higher perceived stress. Consistent with prior research linking class-based discrimination and stigma to poorer mental health outcomes [4,5,14,26], both experienced and anticipated classism were associated with higher levels of depression and anxiety symptoms. Whereas much of the existing literature has relied on specific or higher-risk populations, the current findings conceptually replicate prior work by demonstrating similar patterns in an online census-matched sample of U.S. adults. Extending this literature, confirmatory analyses indicated that perceived stress statistically mediated the relationship between both forms of classism and mental health symptoms. However, the magnitude of the indirect effects was small (υ = 0.01 to 0.03), indicating that only a small portion of the association between classism and mental health outcomes is statistically explained by perceived stress. It is possible there are additional factors not examined in the present study that may also statistically mediate the association between classism and mental health symptoms. These findings offer preliminary support for theoretical models that conceptualize psychological stress appraisal as a potential mediator through which discriminatory experiences influence mental health symptoms in the context of classism. Although the effect size for perceived stress was small, modest-stress related effects may accumulate over time, potentially contributing to meaningful differences in mental health at the population level. Importantly, the direct effects of classism on both depression and anxiety symptoms remained significant after accounting for perceived stress. This suggests there may be additional correlates associated with class-based discrimination with mental health. Experiences of classism may also evoke shame, rumination, or internalized classism, all of which may independently be associated with mental health outcomes. Future research should examine these additional mechanisms to more fully characterize the processes through which classism contributes to mental health disparities.

In exploratory analyses, individuals living at or below the federal poverty line had a higher predicted probability of reporting experienced classism compared with those living above the poverty line. Although preliminary, these findings are consistent with prior work suggesting that exposure to classism may be socioeconomically stratified [2]. Because individuals living at or below the federal poverty line had a higher predicted probability of reporting experienced classism, we conducted additional exploratory analyses within this subgroup. Among individuals living in poverty, experienced classism was associated with higher perceived stress, which in turn was associated with higher depression and anxiety symptoms. This pattern was not observed for anticipated classism. One possible explanation for this pattern is that overt experiences of classism represent salient distal stressors among individuals facing economic disadvantage. This disparity in experienced classism may reflect differences in social contexts characterized by social-evaluative threat and class-based devaluation. Individuals living in poverty may navigate environments where social class cues are more visible in everyday interactions, such as applying for and using federal assistance programs, resulting in greater exposure to class-based discrimination and mistreatment. In contrast, predicted probabilities of anticipated classism appeared similar across poverty status groups, suggesting that concerns about future class-based mistreatment may not be exclusive to those living in poverty. Rather, it is possible that anticipated classism is related to concerns about potential status threat and status loss, which may be experienced across the socioeconomic gradient [9,27]. Future research is needed to directly examine how experienced and anticipated classism are distributed across social contexts and economic groups, to identify the settings in which class-based mistreatment is most likely to occur, and to determine whether these forms of classism are associated with distinct psychological and physiological variables.

Several strengths of the study should be noted. First, the study advances the literature on classism by examining a theoretically grounded psychological mediator linking class-based discrimination to mental health symptoms. The findings provide one of the first empirical tests of psychological stress appraisal processes within the context of classism, extending theoretical models of stigma and identity threat to social class [7]. Second, the use of a census-matched U.S. sample improves the demographic diversity of the sample relative to convenience samples and allows these associations to be examined across a broader range of sociodemographic groups. Third, the study distinguished between experienced and anticipated classism, allowing for an examination of how overt and anticipatory forms of class-based discrimination are differentially associated with mental health outcomes. Fourth, the analyses statistically accounted for objective indicators of socioeconomic status, including income and education. This does not suggest that socioeconomic variables are unrelated to mental health symptoms, but rather that class-based discrimination may be an additional psychosocial factor that is associated with mental health symptoms. Lastly, missing data and mediation analyses were conducted using a Bayesian framework, which incorporated imputation procedures to reduce bias that may arise from a listwise deletion approach.

Results should be interpreted considering several limitations. First, the data were cross-sectional, which limits conclusions regarding the temporal ordering of variables, the direction of associations, and causality. Although the models were theoretically specified such that classism would precede perceived stress and mental health symptoms, it is possible that mental health symptoms influence perceptions of classism and stress. In addition, mediation models estimated with cross-sectional data may be susceptible to common method variance, or systematic error that arises when variables are measured using similar methods at the same time, which can inflate associations and overestimate indirect effects [28]. The current findings should thus be interpreted as statistical mediation rather than evidence for causal mechanisms. Future research should use longitudinal designs to examine mediating processes following exposure to classism and prior to the onset of mental health symptoms to better establish temporal precedence. Second, experienced and anticipated classism were assessed using single-item measures, which limits the breadth and precision of the focal constructs. Single-item measures do not capture the multidimensional nature of classism, particularly when distinguishing between experienced and anticipated forms of classism. Furthermore, the reliability of these measures cannot be evaluated, raising the potential for measurement error that may attenuate estimates. Future research should employ multi-item, validated measures of classism [29] to more comprehensively assess these constructs and their relationships. Third, limitations related to online sampling should be considered. The current study did not include individuals without internet access or non-English speaking participants, which may limit generalizability. Future research could address these gaps by using mixed-methods approaches and oversampling underrepresented groups to improve the generalizability of these findings. Fourth, mental health symptoms were assessed using self-report measures, which are susceptible to reporting bias [30]. Future studies could replicate these findings using clinical diagnostic interviews. Although the current study focused on symptoms to enhance generalizability to non-clinical populations, incorporating diagnostic assessments would allow researchers to extend this work to individuals with clinically significant mental health conditions. Finally, the exploratory analyses were not pre-registered and should be interpreted with caution. Although a consistent analytic approach was used to examine the hypothesis among individuals living in poverty, future research should seek to replicate these findings using pre-registered designs and samples specifically powered to examine processes among economically disadvantaged populations.

## 5. Conclusions

Like other forms of discrimination and stigma, classism is positively correlated with stress and associated with poor mental health symptoms. However, the observed mediation effects were small, indicating that perceived stress explained only a fraction of the association between classism and mental health symptoms. These findings should therefore be interpreted as preliminary evidence of one potential statistical mediator rather than definitive evidence for perceived stress as an underlying mechanism. Future work may consider examining additional psychological or biological factors, including biomarkers of physiological stress (e.g., cortisol), alongside perceived stress to further assess the negative health correlates of classism. Muscatell and colleagues [31] have proposed a model in which racism negatively impacts health through multiple neural and physiological pathways; a similar framework may be useful for understanding class-based discrimination. Ultimately, these findings highlight the need for a broader, multidimensional approach that considers biopsychosocial factors in the context of classism. Future research using longitudinal and experimental designs will be necessary to clarify the temporal dynamics of these associations and to identify additional constructs related to classism and mental health disparities.

## Figures and Tables

**Table 1:** Sample characteristics.

Characteristic	*n*	%
Gender		
Woman	1023	51.33%
Man	965	48.42%
Non-binary/Other term	5	0.25%
Race/Ethnicity		
Asian/Asian-American	103	5.17%
Black/African American	263	13.20%
Hispanic/Latino (a)	309	15.50%
Indigenous, Alaskan Native, or Aleut	25	1.25%
Native Hawaiian/Pacific Islander	2	0.10%
White	1255	62.97%
Biracial/Multiracial	28	1.40%
Other	8	0.40%
Education level		
1	832	47.11%
2	668	37.83%
3	266	15.06%
Income		
Less than $25,000	362	18.16%
$25,000–$49,999	448	22.48%
$50,000–$74,999	384	19.27%
$75,000–$99,999	288	14.45%
$100,000–$149,999	289	14.50%
$150,000–$199,999	112	5.62%
Greater than $200,000	110	5.52%
Federal Poverty Line (FPL)		
Above FPL	1460	82.91%
Below FPL	301	17.09%
Region		
Northeast	357	17.91%
South	732	36.73%
Midwest	453	22.73%
West	451	22.63%

*Note.* Frequencies and percentages were calculated using non-imputed data. Missing data for education level (*n* = 236) and poverty status (*n* = 232). Income is presented as a categorical variable in the table but is included as a continuous variable in mediation models. Education 1 = High school diploma or less/Some college; Education 2 = Associate or Bachelor’s degree; Education 3 = Graduate Degree.

**Table 2:** Descriptive statistics and zero-order correlations between focal variables.

Measure	1	2	3	4	5	6	7
1. Age	--						
2. Poverty Status	**–0.05** *	--					
3. Experienced Classism	**−0.27** ***	**0.07** **	--				
4. Anticipated Classism	**−0.33** ***	**0.07** **	**0.73** ***	--			
5. Perceived Stress	**−0.32** ***	**0.14** ***	**0.31** ***	**0.35** ***	--		
6. Depressive Symptoms	**−0.33** ***	**0.13** ***	**0.42** ***	**0.44** ***	**0.71** ***	--	
7. Anxiety Symptoms	**−0.36** ***	**0.11** ***	**0.45** ***	**0.48** ***	**0.67** ***	**0.81** ***	--
Mean *(SD)*	47.2 (17.3)	0.17 (0.38)	1.69 (0.89)	1.89 (0.96)	2.66 (0.83)	1.17 (1.12)	2.13 (1.05)
Range	18–87	0–1	1–4	1–4	1–5	0–4	1–5
Missingness	0%	11.6%	7.63%	7.63%	7.43%	8.08%	8.48%

*Note.* Values represent Pearson correlation coefficients. Zero-order correlations and descriptive statistics were calculated using non-imputed data. Means, standard deviations (*SD*), ranges, and percentage of missing data are reported for each variable of interest. Bold indicates statistical significance, * *p* < 0.05; ** *p* < 0.01; *** *p* < 0.001.

**Table 3:** Results from the mediation model with full sample.

Predictor	Effect	Depression Symptoms Estimate (*SE*) 95% CI	% Mediated	υ	Anxiety Symptoms Estimate (*SE*) 95% CI	% Mediated	υ
Experienced Classism	Total	**0.30 (0.05) [0.21, 0.39]**	--		**0.31 (0.04) [0.23, 0.40]**	--	
	Direct	**0.20 (0.04) [0.13, 0.28]**	--		**0.23 (0.04) [0.16, 0.30]**	--	
	Indirect	**0.09 (0.03) [0.04, 0.15]**	**30.0%**	**0.01**	**0.08 (0.03) [0.04, 0.13]**	**25.8%**	**0.01**
Anticipated Classism	Total	**0.31 (0.05) [0.22, 0.41]**	--		**0.34 (0.05) [0.25, 0.43]**	--	
	Direct	**0.14 (0.03) [0.07, 0.22]**	--		**0.19 (0.03) [0.12, 0.27]**	--	
	Indirect	**0.17 (0.03) [0.11, 0.23]**	**54.8%**	**0.03**	**0.15 (0.03) [0.10, 0.20]**	**44.1%**	**0.02**

*Note.* Results indicated that the indirect effect of perceived stress was significant. The total effect reflects the overall association between classism and mental health symptoms, without adjusting for perceived stress. The direct effect represents the association between classism and mental health symptoms after accounting for perceived stress. The indirect effect represents the portion of the association statistically explained by perceived stress. The percentage mediated was calculated by dividing the indirect effect by the total effect. Upsilon (υ) represents the squared standardized indirect effect and is interpreted using Cohen’s benchmarks for effect sizes [25]. Bold indicates a significant effect (i.e., the 95% CI does not contain zero). CI = credible interval; *SE* = standard error.

**Table 4:** Mediation model results with poverty subsample.

Predictor	Effect	Depression Symptoms Estimate (*SE*) 95% CI	% Mediated	υ	Anxiety Symptoms Estimate (*SE*) 95% CI	% Mediated	υ
Experienced Classism	Total	**0.31 (0.11) [0.09, 0.52]**	--		**0.22 (0.11) [0.02, 0.43]**	--	
	Direct	**0.19 (0.09) [0.01, 0.36]**	--		0.10 (0.08) [−0.07, 0.27]	--	
	Indirect	**0.12 (0.06) [0.004, 0.25]**	**38.7%**	**0.01**	**0.12 (0.06) [0.004, 0.24]**	**54.5%**	**0.01**
Anticipated Classism	Total	0.20 (0.11) [−0.003, 0.41]	--		**0.30 (0.11) [0.09, 0.51]**	--	
	Direct	0.11 (0.09) [−0.06, 0.28]	--		**0.22 (0.09) [0.05, 0.39]**	--	
	Indirect	0.08 (0.06) [−0.03, 0.21]	--		0.08 (0.06) [−0.03, 0.21]	--	

*Note.* Results indicated that the indirect effect of perceived stress was significant, but only for experienced classism. Among individuals living at or below the federal poverty line, higher experienced classism was associated with higher perceived stress, and higher perceived stress was associated with higher depression and anxiety symptoms. The indirect effect represents the portion of the association statistically explained by perceived stress. The percentage mediated was calculated by dividing the indirect effect by the total effect. Upsilon (υ) represents the squared standardized indirect effect (25). Bold indicates a significant effect (i.e., the 95% CI does not contain zero). CI = credible interval; *SE* = standard error.

## Data Availability

De-identified data and analytic code sufficient to reproduce the results reported in this manuscript are available at the Open Science Framewor (https://osf.io/cvtxh/overview?view_only=31f017e0bf1343039269a66f7a3ed3bc, accessed on 28 April 2026).

## References

[1] Langhout R.D., Drake P., Rosselli F. (2009). Classism in the University Setting: Examining Student Antecedents and Outcomes. J. Divers. High. Educ..

[2] Jokela M., Fuller-Rowell T.E. (2022). Changing Associations between Socioeconomic Status and Self-Reported Discrimination from the 1990s to the 2010s in the United States. Int. J. Psychol..

[3] Figueroa D.G., Parker J.E., Hunger J.M., Kraus M.W., Muscatell K.A., Tomiyama A.J. (2024). Social Class Stigma and Poorer Health Behaviors: Evidence from the Eating in America Study. Soc. Sci. Med..

[4] Inglis G., Jenkins P., McHardy F., Sosu E., Wilson C. (2022). Poverty Stigma, Mental Health, and Well-Being: A Rapid Review and Synthesis of Quantitative and Qualitative Research. J. Community Appl. Soc. Psychol..

[5] Cavalhieri K.E., Wilcox M.M. (2022). The Compounded Effects of Classism and Racism on Mental Health Outcomes for African Americans. J. Couns. Psychol..

[6] Cohen S., Kamarck T., Mermelstein R. (1983). A Global Measure of Perceived Stress. J. Health Soc. Behav..

[7] Major B., O’Brien L.T. (2005). The Social Psychology of Stigma. Annu. Rev. Psychol..

[8] Schneiderman N., Ironson G., Siegel S.D. (2005). Stress and Health: Psychological, Behavioral, and Biological Determinants. Annu. Rev. Clin. Psychol..

[9] Manstead A.S.R. (2018). The Psychology of Social Class: How Socioeconomic Status Impacts Thought, Feelings, and Behaviour. Br. J. Soc. Psychol..

[10] Clark R., Anderson N.B., Clark V.R., Williams D.R. (1999). Racism as a Stressor for African Americans: A Biopsychosocial Model. Am. Psychol..

[11] Pascoe E.A., Richman L.S. (2009). Perceived Discrimination and Health: A Meta-Analytic Review. Psychol. Bull..

[12] Schmitt M.T., Postmes T., Branscombe N.R., Garcia A. (2014). The Consequences of Perceived Discrimination for Psychological Well-Being: A Meta-Analytic Review. Psychol. Bull..

[13] Williams D.R., Lawrence J.A., Davis B.A., Vu C. (2019). Understanding How Discrimination Can Affect Health. Health Serv. Res..

[14] Cavalhieri K.E., Willyard A., Phillippi J.C. (2023). The Effects of Different Types of Classism on Psychological Outcomes: Preliminary Findings. Int. J. Adv. Couns..

[15] Fuller-Rowell T.E., Evans G.W., Ong A.D. (2012). Poverty and Health: The Mediating Role of Perceived Discrimination. Psychol. Sci..

[16] Santiago C.D.C., Wadsworth M.E., Stump J. (2011). Socioeconomic Status, Neighborhood Disadvantage, and Poverty-Related Stress: Prospective Effects on Psychological Syndromes among Diverse Low-Income Families. J. Econ. Psychol..

[17] Marbin D. (2022). Perspectives in Poverty and Mental Health. Front. Public Health.

[18] Williams D.R., Yu Y., Jackson J.S., Anderson N.B. (1997). Racial Differences in Physical and Mental Health Socio-Economic Status, Stress and Discrimination. J. Health Psychol..

[19] Hunger J.M., Major B., Blodorn A., Miller C.T. (2015). Weighed Down by Stigma: How Weight-Based Social Identity Threat Contributes to Weight Gain and Poor Health. Soc. Personal. Psychol. Compass.

[20] Choi S.W., Reise S.P., Pilkonis P.A., Hays R.D., Cella D. (2010). Efficiency of Static and Computer Adaptive Short Forms Compared to Full-Length Measures of Depressive Symptoms. Qual. Life Res..

[21] Kroenke K., Yu Z., Wu J., Kean J., Monahan P.O. (2014). Operating Characteristics of PROMIS Four-Item Depression and Anxiety Scales in Primary Care Patients with Chronic Pain. Pain Med..

[22] Enders C.K., Du H., Keller B.T. (2020). A Model-Based Imputation Procedure for Multilevel Regression Models with Random Coefficients, Interaction Effects, and Nonlinear Terms. Psychol. Methods.

[23] Gelman A., Rubin D.B. (1992). Inference from Iterative Simulation Using Multiple Sequences. Stat. Sci..

[24] Lachowicz M.J., Preacher K.J., Kelley K. (2018). A Novel Measure of Effect Size for Mediation Analysis. Psychol. Methods.

[25] Cohen J. (1988). Statistical Power Analysis for the Behavioral Sciences.

[26] Inglis G., Sosu E., McHardy F., Witteveen I., Jenkins P., Knifton L. (2024). Testing the Associations between Poverty Stigma and Mental Health: The Role of Received Stigma and Perceived Structural Stigma. Int. J. Soc. Psychiatry.

[27] Gruenewald T.L., Kemeny M.E., Aziz N. (2006). Subjective Social Status Moderates Cortisol Responses to Social Threat. Brain Behav. Immun..

[28] Brannick M.T., Chan D., Conway J.M., Lance C.E., Spector P.E. (2010). What Is Method Variance and How Can We Cope with It? A Panel Discussion. Organ. Res. Methods.

[29] Cavalhieri K.E., Chwalisz K. (2020). Development and Initial Validation of the Perceived Classism Experiences Scale. Couns. Psychol..

[30] Latkin C.A., Edwards C., Davey-Rothwell M.A., Tobin K.E. (2017). The Relationship between Social Desirability Bias and Self-Reports of Health, Substance Use, and Social Network Factors among Urban Substance Users in Baltimore, Maryland. Addict. Behav..

[31] Muscatell K.A., Alvarez G.M., Bonar A.S., Cardenas M.N., Galvan M.J., Merritt C.C., Starks M.D. (2022). Brain–Body Pathways Linking Racism and Health. Am. Psychol..

